# A Pattern Theory of Self

**DOI:** 10.3389/fnhum.2013.00443

**Published:** 2013-08-01

**Authors:** Shaun Gallagher

**Affiliations:** ^1^Department of Philosophy, University of Memphis, Memphis, TN, USA; ^2^School of Humanities, University of Hertfordshire, Hatfield, Hertfordshire, UK

**Keywords:** self, pattern theory, cortical midline structures, first-person perspective

## Abstract

I argue for a pattern theory of self as a useful way to organize an interdisciplinary approach to discussions of what constitutes a self. According to the pattern theory, a self is constituted by a number of characteristic features or aspects that may include minimal embodied, minimal experiential, affective, intersubjective, psychological/cognitive, narrative, extended, and situated aspects. A pattern theory of self helps to clarify various interpretations of self as compatible or commensurable instead of thinking them in opposition, and it helps to show how various aspects of self may be related across certain dimensions. I also suggest that a pattern theory of self can help to adjudicate (or at least map the differences) between the idea that the self correlates to self-referential processing in the cortical midline structures of the brain and other narrower or wider conceptions of self.

## Introduction: Variations on the Self

From a philosophical perspective, any claim to explain something called “the self” immediately raises a host of problems. On the one hand, although many philosophers are perfectly comfortable talking about “the self,” what they have to say about this concept usually turns out to be controversial. For example, that the self is socially constructed (Gergen, 2011) or a product of narrative (Schechtman, 2011), and nothing more; that the self is strictly minimal, on the order of 3 s in duration, and nothing more (Strawson, 1999a); that the self as such doesn’t exist at all, plus a lot more about a replacement concept called a “self model” (Metzinger, 2003). Such deflationary and reductionist accounts tend to be reactions against something like a traditional Cartesian notion of the self as a substantial (soul-like) entity, and some of them can be understood as variously inspired by Humean, Buddhist, or neuroscientific perspectives.

On the other hand, and pursuing a different strategy, some philosophers prefer to avoid the phrase “the self” by pluralizing it with important modifiers between “the” and “self.” Thus we find a multitude of variations, once cataloged, with references, by Strawson (1999b) as follows:

[T]he cognitive self, the conceptual self, the contextualized self, the core self, the dialogic self, the ecological self, the embodied self, the emergent self, the empirical self, the existential self, the extended self, the fictional self, the full-grown self, the interpersonal self, the material self, the narrative self, the philosophical self, the physical self, the private self, the representational self, the rock bottom essential self, the semiotic self, the social self, the transparent self, and the verbal self (cf. e.g., James, 1890; Stern, 1985; Dennett, 1991; Gibson, 1993; Neisser, 1994; Cole, 1997; Butterworth, 1998; Gazzaniga, 1998; Legerstee, 1998; Gallagher and Marcel, 1999; Pickering, 1999; Sheets-Johnstone, 1999).

Trying to improve on this list would likely lead to nitpicking about terms, but we may want to add “the neural self,” “the synaptic self” (LeDoux, 2002); or what we might call “the midline self” [in reference to self-referential processes in the cortical midline structures (CMS) (Northoff and Bermpohl, 2004)]. The list of variations is likely not complete. Someone might think that the question is: “Which is it?” – which one is *the* self? Or perhaps, which one is the primary meaning of self? It’s not clear, however, that one has to choose just one variation. Many of these concepts of self were developed in the plural. James (1890), for example, distinguished between the physical self, the social self, and the private self. Neisser (1988) discussed five types of self-knowledge corresponding to the ecological self, the interpersonal self, the conceptual self, the extended self, and the private self. Despite the terminology suggesting a plurality of selves, however, Neisser (1991) carefully refers to them as aspects of self – e.g., the ecological aspect of self.

In this paper I propose to stay plural about the concept of self, and to follow Neisser’s more careful vocabulary referencing different aspects of self. In this regard, however, I want to argue that we should not think of such aspects as aspects *of* “the self,” as if they are simply modifying something that has its own independent existence. Rather, I propose that we think of these aspects as organized in certain patterns, and that a particular variation of such a pattern constitutes what we call a self. In the following sections I’ll try to make this idea clear, and I’ll try to indicate some advantages of thinking of self in this way.

In part, this approach is motivated by various issues that relate to the theory of self as involving CMS and self-referential processing, as developed by Northoff and others (Northoff and Bermpohl, 2004; Northoff et al., 2006). Some critical studies, for example, have suggested that in terms of brain processes, the self is both everywhere and nowhere in the brain (Gillihan and Farah, 2005; Vogeley and Gallagher, 2011). Others challenge the idea that the self correlates to CMS processing, and argue that such processes are not self-specific because activation in these areas also corresponds to non-self discrimination (Legrand and Ruby, 2009). Although I think some of these criticisms raise important points, I argue here that midline processes do tell us something important about the notion of self and may correlate with specific aspects that are part of the pattern that we call self.

## Pattern Theories

Let me first say that in talking about pattern theories I do not mean to associate a pattern theory of self with “Pattern Theory” in mathematics (Grenanderm, 1994). This kind of mathematical formalism may or may not be a helpful tool for the analysis of the specific patterns that I will discuss. I remain neutral on that point. In any case, one can understand the notion of pattern at stake here without having to understand Pattern Theory in this sense. Furthermore, although there are numerous theories that are referred to as “pattern theories,” e.g., pattern theory of pain (Goldscheider, 1894; Sinclair, 1955), dynamic pattern theory of motor control (Kelso, 1995), etc. these theories don’t necessarily share the same general principles, and at the most general level the concepts of pattern represented in the different theories may be incommensurable with each other. Accordingly, since, for purposes of economy I want to avoid starting from scratch in developing a pattern theory of self, I will follow a strategy that allows me to point to an already established theory, one that can operate as a heuristic model for our purposes – i.e., one in which the concept of pattern is used in a way that is not incommensurable with what I take to be the pattern theory of self. Although we could think of psychological discussions of pattern recognition as a kindred notion, more specifically I suggest that we consider what I’ll call a pattern theory of emotion to be a good model for a pattern theory of self. There are two reasons why a pattern theory of emotion may be a good model in this regard: (1) it reflects a commensurable concept of pattern (i.e., it refers to the same kind of pattern that I think is relevant to the notion of self, and (2) it may contribute directly to a pattern theory of self since, as I’ll suggest, affect is one aspect that forms part of the pattern of self.

The pattern theory of emotion claims that emotions are complex patterns of bodily processes, experiences, expressions, behaviors and actions, and as such they are “individuated in patterns of characteristic features” (Izard, 1972; Izard et al., 2000; Mendoça, 2012; Newen et al., under review). On a pattern theory, “emotion” is a cluster concept that includes a sufficient number of characteristic features. Taken together, a certain pattern of characteristic features constitutes an emotion, although no individual feature by itself may be necessary to constitute an emotion. This means, as Newen et al. (under review) point out, there are borderline cases where it is not clear whether some complex cluster of aspects counts as an emotion.

Izard et al. (2000) develop this idea under the title of differential emotions theory (DET), maintaining that emotions operate as complex systems that emerge from dynamic interactions of constituent neuro-hormonal, motoric, and experiential processes (Izard, 1972). Emotion patterns draw from components that are set up as evolutionary adaptations. In the emergence of any particular emotion, however, organism-environment transactions play a role. Individual emotions may also combine or co-assemble with other emotions to form new emotion patterns that may stabilize over repeating occurrences. On this view, discrete emotions are dynamically self-organizing in that “recursive interactions among component processes generate emergent properties” (Izard et al., 2000, p. 15). Different emotions are constituted by different patterns of processes that yield behavioral performances that vary from one individual to another, and within individuals over time. Importantly, such behaviors should not be regarded simply as an expression of an emotion, but rather are part (an emerging feature) of the pattern that constitutes the emotion.

Newen et al. (under review) provide a catalog of different features that may contribute to specific patterns that constitute emotions. They include:

(1) Autonomic processes: one might think of James’ (1884) claim that an emotion is the perception of bodily changes that include autonomic nervous system (ANS) activity. For a pattern theory of emotion autonomic activity is only one possible constituent, and it may be perceived (experienced) or not. Not every emotion has a distinct ANS pattern, and different emotions need not have different ANS patterns (Prinz, 2004).
(2) Actions: including what Frijda calls “action tendencies,” bodily changes preparatory for actions that may be experienced as urges to perform a certain kind of action (Frijda, 1986). Some emotions, e.g., happiness, may not include this component; others may be typically associated with specific actions (e.g., freezing or fleeing in fear).
(3) Overt expressions: including expressive posture and movement, facial expression, gesture, and vocal expressions (e.g., intonations, screams, laughter). Such individual expressions may themselves combine into a typical emotion-related pattern themselves.
(4) Phenomenal feeling: this conscious or experiential component is often part of an emotion, although it is not necessary for every emotional occurrence. In some rare cases typical physiological, expressive, and cognitive aspects may be present without the phenomenal aspect (e.g., in those disposed to repress fear (Sparks et al., 1999).
(5) Cognitive aspects: such as attitudes, shifts of attention, and changes to perception. Cognitive attitudes may include, for example, as Newen et al. suggest, belittling thoughts about one’s rival in the case of jealousy or a judgment that one has been treated unfairly in certain cases of anger. Such attitudes may or may not be manifested in behavior or in verbal reports. Shifts of attention, may include, for example, being alerted to specific aspects of the environment in the case of fear. Affect is an essential aspect of perception (Bower and Gallagher, in press) and emotions can make us notice things we otherwise would not have noticed or can motivate us to see things a certain way.
(6) Intentional objects: that is, the perceived, remembered or imagined object the emotion is about. Newen et al. quote (Goldie, 2000, pp. 16–17). “This can be a particular thing or person (that pudding, this man), an event or an action (the earthquake, your hitting me), or a state of affairs (my being in an aeroplane).”
  I would add to this list:
(7) Situational aspects: following Dewey, who, in his critique of James, points out that emotions are not reducible to a set of bodily states, but also, since the body is always coupled to an environment, always include situational aspects. The unit of analysis should always be organism-environment. Situational aspects, and the fact that emotional experiences and behaviors are always situated, are part of the pattern (Mendoça, 2012, 2013). In this regard it is not just the intentional object, but also the situation reflected in the intentional structure of the emotion, that helps to disambiguate emotional expressions. Importantly, situations are almost always social and/or cultural and such factors contribute constitutively to what an emotion is.

Such aspects are variables that can take different values and weights in the dynamic constitution of an emotion. Some values are more or less likely to occur together. In this respect we can distinguish typical patterns of aspects and values and define an emotion as involving some variation of that pattern. Newen et al. are careful to note that to say a particular feature is constitutive of an emotion does not mean that it is an essential component. On the pattern theory of emotion such features are not constitutive in the essentialist sense. One can have a token of the same type of emotion lacking a particular characteristic feature, although there may be some minimal number of characteristic features and their values that are sufficient to constitute a particular pattern that counts as that emotion.

A feature f is constitutive for a pattern X if it is part of at least one set of features which is minimally sufficient for a token to belong to a type X. “Minimally sufficient” means that these features are jointly sufficient for the episode to be of type X, but if one of them would be taken away the episode would not count as a instance of type X anymore (Newen et al., under review).

It is possible, of course, to include other aspects or characteristics in the list above. One may want to include more than just autonomic processes under a broad heading of embodied processes, for example. One may want to list certain brain patterns as part of an emotion pattern. I think, however, that the list provides sufficient detail to indicate the kind of pattern theory that we want to consider. Let me just note that one of the advantages of this theory of emotion is that it becomes very easy to say that we can perceive emotions in others. If emotions are constituted by features that may include bodily expressions, behaviors, action expressions, etc., then emotion perception can be considered a form of pattern recognition (Newen et al., under review; Gallagher and Varga, in press).

## A Pattern Theory of Self

In a way similar to the construction of a pattern theory of emotion, I want to suggest that we can develop a pattern theory of self. On such a view, what we call self consists of a complex and sufficient pattern of certain contributories, none of which on their own is necessary or essential to any particular self. This is not a pattern theory of “*the* self.” Rather, what we call “self” is a cluster concept which includes a sufficient number of characteristic features. Taken together, a certain pattern of characteristic features constitute an individual self. It seems possible that this would allow us to identify borderline cases where it is not clear whether some complex cluster of aspects would count as a self – here one might think of Dissociative Identity Disorder and the idea that there may be more than one self involved in such cases. On this view selves operate as complex systems that emerge from dynamic interactions of constituent aspects. It may also be the case that self-patterns draw from components that, like the components of emotion, are set up as evolutionary adaptations. Indeed, emotion-related aspects may contribute to the constitution of a self^1^. Different selves are constituted by different patterns, but within one individual these patterns may change over time.

One important issue concerns the level of analysis at which we put the pattern theory of self to work. There are three possible levels to think about. First, one can think of the pattern theory of self as operating like a meta-theory that defines a schema of possible theories of self, each of which would itself be a pattern theory. For example, the meta-theory can claim that elements *a* through *g* are all possible aspects that can be included in any particular pattern theory of self. Such a meta-theory would aim to provide a complete list of such elements and to map out all possible pattern theories of self. Accordingly, at this level there would be no claims made about necessary or sufficient conditions for constituting a self. Second, however, any particular theory of self can be a pattern theory, and one pattern theory can differ from another pattern theory by specifying different aspects (from among *a* through *g*) to be included as aspects of self. In this respect, one can think of a pattern theory of self as defining the self at the level of a type, and at this level the theory might specify necessary or sufficient conditions, indicating, for example, that *a* and *b* are necessary but not sufficient for selfhood. Finally, however, one can think that in any particular instance, at the level of a particular token, a pattern theory of self can apply to an individual self. A particular self may manifest or include a pattern of only aspects *a* through *d* and be considered a self even if all aspects defined by the relevant pattern theory of self are not included. The analysis in this paper remains on the meta-theoretical level unless otherwise noted.

What features can contribute to specific patterns that constitute a self? To philosophers it will come as no surprise that what gets included in this list is open to contentious debate. Keep in mind, however, that, remaining at the level of meta-theory, we are not talking about necessary conditions. A particular theory of self may exclude some of these conditions, and a particular self may lack a particular characteristic feature as defined here and still be considered a self. Here is a tentative list. I do not claim that it is complete. Under each heading I offer some un-systematic notes to indicate the scope of each aspect (or set of aspects).

(1) Minimal embodied aspects: include here core biological, ecological aspects, which allow the system to distinguish between itself and what is not itself. This is an extremely basic aspect of all kinds of animal behavior. One should also include those aspects that define the egocentric (body-centered) spatial frame of reference, which reflects a first-person perspective, and contributes to specifications of possible actions in peripersonal space.
(2) Minimal experiential aspects: to the extent that the bodily system can be conscious, it will pre-reflectively experience, from a first-person perspective, the self/non-self distinction in the various sensory-motor modalities available to it (e.g., kinesthesia, proprioception, touch, vision). Such aspects contribute to an experiential and embodied sense of ownership (the “mineness” of one’s experience, as well as of one’s body and movement), and a sense of agency for one’s actions (Gallagher, 2000, 2012a; Rochat, 2011).
(3) Affective aspects: the fact that someone manifests a certain temperament may reflect a particular mix of affective factors that range from very basic and mostly covert or tacit bodily affects to what may be for her a typical emotional pattern or mood, For example, someone may be a typical extrovert who enthusiastically engages in outwardly directed actions.
(4) Intersubjective aspects: human and possibly some non-human animals are born with a capacity for attuning to intersubjective existence (Neisser, 1988); this may take the form of being aware that someone else is present and possibly gazing at you. Human infants attend to the gaze and the eye direction of others. There is a certain point in such situations where a more developed self-consciousness arises – a sense of self-for-others (Sartre, 1956); a self-conscious recognition of oneself as being oneself as distinct from others. This is sometimes associated with mirror self-recognition (see Gallup et al., 2011). Mead (1913) famously suggested that the self (in this developed sense) originates in such intersubjective/social interactions. Others suggest that in those systems capable of language, this intersubjective aspect is internalized and takes the form of a dialogical process which helps to constitute the self (see Hermans, 2011).
(5) Psychological/cognitive aspects: traditional theories of the self focus on these aspects, which may range from explicit self-consciousness to conceptual understanding of self as self, to personality traits of which one may not be self-conscious at all. In addition, there are strong arguments for psychological continuity and the importance of memory in the literature on personal identity (e.g., Shoemaker, 2011). Most often philosophers think of these aspects as part of a private, internal kind of existence; neuroscientists may characterize these aspects in terms of neuronal processes. One might also include representational aspects here, where this means something like one’s ability to represent oneself as oneself (to oneself, but also perhaps to others).
(6) Narrative aspects: although there are many variations of this idea, the basic claim is that selves are inherently narrative entities (Schechtman, 2011), and for some theorists, narratives are constitutive for selves. Our self-interpretations have a narrative structure. On some views it is important that narratives are generated by the brain, a fact that leads some to consider narratives mostly as fictions (Gazzaniga, 1998) and selves as abstract “centers of narrative gravity” (Dennett, 1991).
(7) Extended aspects: James (1890) suggested that what we call self may include physical pieces of property, such as clothes, homes, and various things that we own. We identify ourselves with stuff we own, and perhaps with the technologies we use, the institutions we work in, or the nation states that we inhabit.
(8) Situated aspects: these are aspects that play some (major or minor) role in shaping who we are. They include the kind of family structure and environment where we grew up; cultural and normative practices that define our way of living, and so on (see Gergen, 2011).

Such aspects are variables that can take different values and weights in the dynamic constitution of a self. This pattern theory of self will not solve all philosophical problems of course. One may want to know which of these aspects are necessary or essential, and this might be specified by a particular pattern theory of self. As such theories get applied to individuals, for example, it seems possible that one may experience life in a less continuous or coherent way than others do, thereby minimizing the narrative aspect, without minimizing the sense of self or self-identity (Strawson, 2004). One may also lose a sense of agency, as in some schizophrenic symptoms, without losing a sense of ownership or other aspects that define a self (Gallagher, 2005). One might lose the ability to recall one’s past life, as in some cases of amnesia and Alzheimer’s disease, and may also undergo character or personality change; in such cases one’s self-identity may continue to be supported by one’s minimal bodily and experiential aspects, as well as by intersubjective relations and/or extended aspects in one’s surroundings. This is not to say that such changes do not result in a modulation of self-experience or self-identity, but rather, since self is not reducible to any one of these aspects, it is a modulation rather than a complete loss. There may be various states of existence or pathologies associated with each of these aspects such that the aspect in question is eliminated or seriously modified.

On the one hand, we can think of a particular pattern theory of self where no one feature is constitutive in an essentialist sense. If someone lacks memory or a sense of agency, or perhaps lacks both, she continues as a self if there are a sufficient number of aspects still intact. On the other hand, we can think of a different particular pattern theory of self where certain aspects are defined as necessary. Beyond such differences, there are still a number of questions outstanding for any particular pattern theory of self. Is there some minimal number of aspects, or some specific combinations of aspects sufficient to constitute a particular pattern that counts as self? Is there a hierarchical relation among these aspects? For example, if someone lacked certain minimal experiential aspects, would their lives still reflect a narrative structure? Different answers to these questions define different variations of a pattern theory of self. It would be difficult to talk of a pattern, or a self, however, if only one aspect is claimed as necessary and sufficient for selfhood. Indeed, if that were the claim, the “aspect” would no longer be an aspect (of a self, or of a pattern); it would *be* the self. The pattern theory of self rules out this kind of reduction, *a priori*, although it does not rule out various answers to the questions mentioned above. At the level of the meta-theory one can also ask: how many different patterns are viable?

## Some Benefits of a Pattern Theory of Self and Its Relevance to CMS Processes

One benefit of the pattern theory of self is that we can more clearly understand various interpretations of self as compatible or commensurable instead of thinking them in opposition. For example, different definitions of personhood can be accommodated or can be viewed as different interpretations that place different weights on some aspects rather than others. If with Locke we define person to mean “a thinking intelligent being, that has reason and reflection, and can consider itself as itself, the same thinking thing, in different times and places; which it does only by that consciousness with is inseparable from thinking, and, as it seems to me, essential to it …” (Locke, 1690/1979, 318), then we can see clearly that this notion of person focuses on psychological aspects of self and ignores other aspects. Other definitions of personhood may emphasize bodily continuity, the importance of social role or legal standing. Differences in definitions of personhood, however, do not necessarily imply differences in definition of self. We may disagree about where to lay the emphasis in defining personhood, but continue to agree that a self is composed of some pattern of aspects, some of which are relevant to the notion of personhood, and others which are not. When we focus on or emphasize a certain pattern or organization of aspects from a certain vantage point (an interpretation which may be tied to social roles, or to causal, legal or moral responsibility, to or certain cultural practices, etc.), we can easily understand self to accommodate different concepts of person, or moral agent, or experiential subject, or physical individual, or mental entity, etc. The pattern theory of self, at the meta-level, remains neutral with respect to these interpretations, and in some respects defines the field of reference or common ground on which such debates about personhood or moral agency or other interpretations of self can take place.

Another advantage is that the pattern theory helps us to see that the various aspects of self may be related in important ways. Many of the particular elements included in the various aspects are themselves complex features of existence that may not be conceptually bound to just one aspect. Thus, for example, the sense of agency in some basic way may be tied to motor control and the sensory-motor operations of the body, but it is also related to social and cultural norms and expectations (which may place limitations on agency) and to psychological/cognitive processes of deliberation and decision-making (Gallagher, 2012a). Something like the sense of agency is interwoven into several aspects of self. To the extent that something like this applies to other elements, then it will be difficult to make the case that there is one and only one aspect that defines self in all cases.

It is in this respect that the pattern theory of self may help to make sense out of some of the controversies surrounding the notion that self is related to cortical midline regions. One claim made in connection with what I’ll call the midline theory of self (or for short, the midline self) is that there is a common element that unites different aspects of self, an integrative glue that holds the pattern together, and that this common element is a processing of stimuli as self-referential (Northoff et al., 2006). The notion of self-referential is then defined in terms of pre-reflective experience, which is found across a diversity of contexts, “autobiographical, social, spatial,” and various others. It is also noted that in any particular case pre-reflective self-referential experience has an affective or emotional dimension. In these regards the notion of self-referential experience includes a number of aspects that can be accommodated by the pattern theory of self. One problem that arises, however, is that pre-reflective experience is extremely difficult to operationalize in experimental settings. Thus Northoff et al. (2006) in discussing experimental data shift the focus to processes that involve reflection or judgment, such as a trait adjective judgment task. For example, in a study by Kelley et al. (2002) subjects are asked to judge whether trait adjectives (e.g., “polite”) more closely described “the participants themselves (self-referential), the current U.S. President (other-referential), or a given case (case-referential)” (Northoff et al., 2006, p. 441). Such experiments activate a variety of brain areas – medial cortex, ventro-, and dorsolateral prefrontal cortex, lateral parietal cortex, bilateral temporal poles, insula, and subcortical regions, including brain stem, colliculi, periaqueductal gray (PAG), and hypothalamus/hypophysis (Northoff et al., 2006, p. 441). Northoff argues that based on a review of recent brain-imaging studies, there are certain core areas commonly activated for self-referential behavior, the so-called CMS. The studies reviewed, however, included only those comparing self- and non-self-related tasks – that is, tasks where subjects had to discriminate between self and non-self – a point that motivated the critique by Legrand and Ruby.

Legrand and Ruby (2009) suggested that there are cognitive processes common to all of the tasks involved in the Northoff et al. review, namely a reflective process of differentiating self and non-self and often involving non-domain specific inferential processing and memory recall. This means that the activated CMS are not dedicated exclusively to self since processes related to non-self, and often to other persons are involved. Indeed, Legrand and Ruby demonstrate “that the main brain regions recruited for others’ mind representation are also and precisely the main brain regions reported in self studies and that this overlap extends beyond the brain areas usually pointed out …” (p. 254). The self-referential processes at stake in these studies are not self-specific in the technical sense proposed by Legrand and Ruby as being (1) exclusively about self (and not about non-self) and (2) non-contingently (i.e., necessary for the process to be) about self. They suggest that only one thing actually meets the self-specificity requirements: the first-person perspectival nature of experience. First-person perspective is exclusively self-related (since it does not apply to the non-self) and non-contingent (since changing or losing the first-person perspective amounts to changing or losing the self–non-self distinction).

On the one hand, Legrand and Ruby want to specify one necessary condition of selfhood; on the other hand, this does not rule out that there are other relevant aspects of self that are important: “We do not claim that all there is to the self can be subsumed under a single process but propose that both basic and complex forms of self have to rely at least partly on self-specific processes …” (2009, p. 279). Whether or not first-person perspective is a necessary condition of selfhood (see, Gallagher, 2012b for a positive answer in agreement with Legrand and Ruby), the disclaimer about subsuming self under a single process is important.

The important move here is to admit that there are multiple processes that may count as self-related, even if not self-specific, and that they can be constitutive of self over and above first-person perspective. That sends us back to a pluralist approach, and it also opens up a theoretical space for the idea that processes associated with CMS, among other aspects, are relevant to what we call self. Indeed, Northoff et al. (2006) (also Northoff et al., 2011; Qin and Northoff, 2011) point to multiple processes that contribute to different aspects of self. These are processes in the verbal domain (as in trait adjective judgment tasks), spatial domain (egocentric vs. allocentric); memory domain (in relation to self-referential information); emotional domain (self-related vs. non-self-related); facial recognition domain (self vs. non-self); social domain (where, according to Northoff et al.’s simulation theory approach, understanding of others depends on self-simulations); and domains that involve agency and ownership. All of these domains have a place within the pattern theory of self. Processes that pertain to memory and face recognition are clearly part of what we referred to as psychological/cognitive aspects. Those that pertain to language (verbal domain) may also be cognitive or may include narrative aspects. Processes pertaining to the emotional domain belong to affective aspects; those that pertain to spatial domain are closely related to first-person perspective, but nicely fit with minimal embodied aspects, while those that pertain to agency and ownership are part of the minimal experiential aspects. Social domain processes are clearly part of the intersubjective aspects. More generally, given that all of these processes reflect a self/non-self matrix, they demonstrate how the minimal embodied aspect of self/non-self differentiation is interwoven into the various other aspects of self. It has also been suggested, however, that minimal experiential aspects of self, connected with basic self-awareness, are interwoven with all other aspects of self, and moreover, that this minimal self-referential awareness survives damage to critical areas in the CMS (Philippi et al., 2012).

Accordingly, the concept of a midline self points to a specific pattern that includes a significant set of interconnected aspects, but not all of the aspects identified in the previous section. The midline theory of self is one particular pattern theory of self. Whether the aspects reflected in self-referential processing in CMS constitute “the core of our self,” as Northoff et al. claim, is of course open to debate. One could go more minimal and claim that the core is, as Legrand and Ruby suggest, a very minimal embodied aspect, or go wider to include aspects that may go beyond CMS related processes, such as extended and situated aspects, or very basic aspects of self-awareness that survive damage to CMS areas (Philippi et al., 2012).

That extended and situated aspects, as well as other aspects included in a pattern theory of self, may enter into a definition of self also suggests an important proviso on the type of approach taken by researchers who are looking specifically at neural processes that reflect these different self-referential behaviors. The patterns at stake in a pattern theory of self are not reducible to neuronal patterns, or patterns of brain activation. This is the case not only for extended and situated aspects, but also for aspects that relate to one’s body, emotional, and intersubjective life, cognitive and narrative dimensions, and so forth. In each case more factors than just brain processes are involved. Although we can expect that brain processes will in some way reflect the way a self is constituted across these different factors, who we are, or what self is, is more than the brain. In this respect, and at the very least, the pattern theory of self helps to map out more precisely what the possibilities are for a non-reductionist, non-deflationary theory of self that is also not inflated into a traditional Cartesian theory of the self as a substantial (soul-like) entity.

## Conflict of Interest Statement

The author declares that the research was conducted in the absence of any commercial or financial relationships that could be construed as a potential conflict of interest.

## Footnotes

^1^In this respect we may start to think of the self as a meta-pattern of various constituent patterns or sets of patterns.
